# Circular Letter to the Members of the Texas State Medical Association

**Published:** 1888-07

**Authors:** J. F. Y. Paine

**Affiliations:** President; Texas State Medical Association, Executive Office, Galveston, Texas


### Society Notes.

### CIRCULAR LETTER TO THE MEMBERS OF THE TEXAS STATE MEDICAL ASSOCIATION.

[Official.]

Texas State Medical Association, Executive Office,
Galveston, Texas, July 16, 1888.

*To the Members of the Texas State Medical Association :*

I have just received official information through the Secretary of the Association that the funds in the treasury are insufficient to pay for the 'publication of the Transaction of the Galveston meeting and the Report of the Committee on Surgical Cases, which is supplemental to the Transactions. I have also been notified through the same official that many members are in *arrears of annual dues*, payment of which would more than discharge all liabilities.

The amount of deficiency is about $250, and unless it be made up, the Annual Volume of Transactions and Surgical Report referred to must either fall below their predecessors in style and completeness, or not issue at all. Both of which contingencies are alike incompatible with the dignity and advancement of the Association, and cannot be permitted to prevail.

The superior merit of the matter comprised in the former numbers of the published transactions has been the means of elevating the Texas State Medical Association to a position in the foremost rank of the similiar institutions of this country, and investing its members with a title which challenges the respect of medical men everywhere. The able Reports of the distinguished Chairman of the Committee on Surgical Cases have created a name for Texas Surgery, fame for her Surgeons, and have received the highest commendations of the medical press of this country and Europe.

The high estate of distinction and usefulness into which the Association has grown, can only be maintained by publishing these Reports, for they are simply the records of the zeal and labor of its members.

A tax of $5 per annum is imposed upon each member, under the provision of Article V of the By-laws, for the support of the Association, compliance with which would create a revenue ample for all purposes whatever. This law, however, has been so *generally overlooked* during the present year as to cause serious financial embarrassment and constrain the Executive to issue this notice. The vital interests of the Association must not be allowed to suffer on account of want of money; and to prevent which, delinquents are earnestly requested to respond promptly to the Secretary's notification, which will issue with the least possible delay; if they should not, a call will be made for voluntary contributions to meet the present exigency.

With the utmost confidence in the patriotism and professional pride of my associates, and a strong faith in their fidelity to a common purpose,

I am very faithfully,

J. F. Y. PAINE, M. D.,
President Tex. S. M. Association.

